# Neutrophil Extracellular Traps Tied to Rheumatoid Arthritis: Points to Ponder

**DOI:** 10.3389/fimmu.2020.578129

**Published:** 2021-01-29

**Authors:** Wenpeng Song, Jing Ye, Nanfang Pan, Chunyu Tan, Martin Herrmann

**Affiliations:** ^1^Department of Rheumatology, West China Hospital of Sichuan University, Chengdu, China; ^2^Department of Internal Medicine 3, Universitätsklinik Erlangen, Friedrich-Alexander-Universität Erlangen-Nürnberg, Erlangen, Germany

**Keywords:** neutrophil extracellular traps, citrullinated autoantigens, anti-citrullinated protein antibodies, autoimmunity, chronic inflammation, protein-arginine deiminase type 4, rheumatoid arthritis

## Abstract

In recent years, neutrophil extracellular traps at the forefront of neutrophil biology have proven to help capture and kill pathogens involved in the inflammatory process. There is growing evidence that persistent neutrophil extracellular traps drive the pathogenesis of autoimmune diseases. In this paper, we summarize the potential of neutrophil extracellular traps to drive the pathogenesis of rheumatoid arthritis and experimental animal models. We also describe the diagnosis and treatment of rheumatoid arthritis in association with neutrophil extracellular traps.

## Introduction

Rheumatoid arthritis (RA) is a chronic inflammatory disease with high disability and increased mortality. It is characterized by progressive joint damage and synovial membrane hypertrophy. There are stark differences in the prevalence among different ethnicities and populations. RA is a heavy burden for the patients, their families, and society. To date, studies have shown that RA is a multifactorial disease involving age, sex, environmental, epigenetic, and genetic factors. However, the pathogenesis of RA is not fully understood (1). Many studies have shown that both innate immune response and adaptive immune response contribute to the etiopathogenesis of RA (2). It’s considered that the formation of autoantibodies to citrullinated antigens (ACPA) is a critical pathogenic event involved in the development of RA. Neutrophils isolated from patients suffering from autoimmune diseases present enhanced formation of neutrophil extracellular traps (NETs). The role of neutrophils in autoimmune disease is still elusive (3). The release of cytotoxic products [e.g., degradation enzymes and reactive oxygen species (ROS)] from activated neutrophils into the synovial fluid and pannus in RA has been known for a long time and is considered important for RA (4, 5). In recent years, it was discovered that neutrophils participate in the inflammatory progression of RA through multiple regulatory immune mechanisms, including directly secreting cytokines and chemokines, and releasing neutrophil granules that activate or inactivate cytokines and chemokines. Enzymes upregulate the expression of MHC II and promote cell-cell interactions.

A novel role of neutrophils, the release of NETs, has attracted increasing attention. Upon pharmacological activation with phorbol myristate acetate (PMA) (6), interleukin 8 (IL-8) (7), or lipopolysaccharide (LPS) (8), neutrophils release granule proteins and chromatin to form NETs. The release of NETs (9) constitutes a novel programmed cell death that differs from apoptosis (10). LPS-induced NET formation increases with adhesion and substrate elasticity, while PMA-induced NET formation is independent of adhesion (6). NETs are composed of chromatin and granular proteins, which trap and kill bacteria (11, 12). Most DNA is derived from nuclei however, mitochondrial DNA is also included. The proteins consist of neutrophil elastase (NE), myeloperoxidase (MPO), histones, defensins, calprotectin (13), matrix metalloproteinase-9, and others (14). During NET formation, NE, MPO, and peptidyl arginine deiminase (PAD4) promote nuclear decondensation and histone citrullination, respectively (15). NETs can be quantified and analyzed by DNA area and NETosis analysis (DANA). Higher frequencies of NETs are detected in subjects with RA (16).

NET formation conventionally occurs *via* the NADPH oxidase (NOX) and ROS-dependent suicidal pathway in which neutrophils rupture and release NETs. Suicidal NETosis is triggered by the engagement of specific receptors or other biomolecules, such as IgG-Fc receptors, Toll-like receptors (TLRs), complement molecules, and cytokines on neutrophils (11, 17–19). The formation of suicidal NETosis is a gradual process that is commonly initiated by the generation of ROS. Then NE and MPO are transported into the nucleus where histones are modified. Finally, nuclear and cellular membranes break, and NETs are released (9, 10, 14). During this process, substantial morphological changes occur. Neutrophils flatten and form membrane protrusions after stimulation (11). Nuclear lobules disappear and chromatin decondense. The inner and outer nuclear membranes detach. The nuclear membrane disintegrates into vesicles, and nuclear material mixes with the cytoplasm to form a homogenous mass. Finally, the neutrophils round up and rupture to release the NETs (10). ROS are pivotal for suicidal NETosis formation (20). ROS are mainly generated by NOX during the “respiratory burst” of neutrophils (21). Patients with chronic granulomatous disease harbor mutations of NOX genes and show reduced NET formation (10, 22). Finally, the NOX complex converts molecular oxygen to hydrogen peroxide, which is a substrate of MPO and is sufficient to induce NET formation (10). The reaction of hydrogen peroxide and MPO can form hypochlorous acid. The latter induces the generation of chlorinated polyamines that may cross-link NET proteins, which maintains the ordered structure of NETs and increases the capacity to trap bacteria (23). MPO partly binds to NE to form the azurosome complex that spans granular membranes without dissolution of the granular membranes (24). NE is a critical enzyme involved in many pathways of NET formation. Methicillin-resistant *Staphylococcus aureus* (MRSA) infected mice with NE deficiency fail to form NETs (25). However, other studies have demonstrated that NE is not required for NET formation induced by noninfectious stimuli; meanwhile, NE deficiency has little effect on histone citrullination (26). NE combines with F-actin filaments to enter the nucleus before MPO. The proteolytic activity of NE is determined by MPO. Patients with mutant MPO also show reduced NET formation (22). As NE translocates from cytoplasm to nucleus, it cleaves histones and participates in chromatin decondensation (27).

In addition to chromatin decondensation performed by NE, another important chromatin modification is histone citrullination driven by PAD4. During NET formation, calcium influx of neutrophils activates a high amount of PAD4, which catalyze histone to citrullinated protein. This enzyme citrullinates arginine residues in the core histones H2A, H3, H4, thus reducing their positive charge, which weakens the interactions between histones and DNA and further promotes chromatin decondensation (28). Five calcium molecules are bound to every PAD4 molecule (29). Interestingly, citrullination driven by PAD4 is inhibited accompanied by inhibition of NOX (28), which may be due to the NOX-induced increase in cytoplasmic calcium levels that activate PAD4 (30). Citrullination driven by PAD4 is induced by LPS and PMA (31). PADI4-deficient mice failed to form NETs after treatment with certain stimuli (32–34).

However, Clark et al. first reported vital NETosis in which neutrophils remained impermeable for SYTOX Green after releasing NETs, which suggested that the neutrophils maintained an intact plasma membrane during NET formation (7). Subsequent researchers found that NETs were induced by blebbing of the nuclear envelope and vesicular exportation in *S. aureus* infection (35). Vital NETosis is activated by pathogen-associated molecular patterns (PAMPs) or endogenous damage-associated molecular patterns (DAMPs). Stimuli recognized by TLR4, such as LPS, may initiate vital NETosis. The nucleus loses its multilobular shape and becomes rounded. Then, the nuclear double membranes vanish, and vesicles composed of DNA filaments bud. These vesicles approach toward the plasma membrane. DNA is released through a small area on the cell surface. Suicidal NETosis and vital NETosis can be distinguished by cleaved N-terminal histone tails (36).

## Roles of NETs in RA

### Pathogenesis of RA Related to NETs Formation

**Figure 1:**
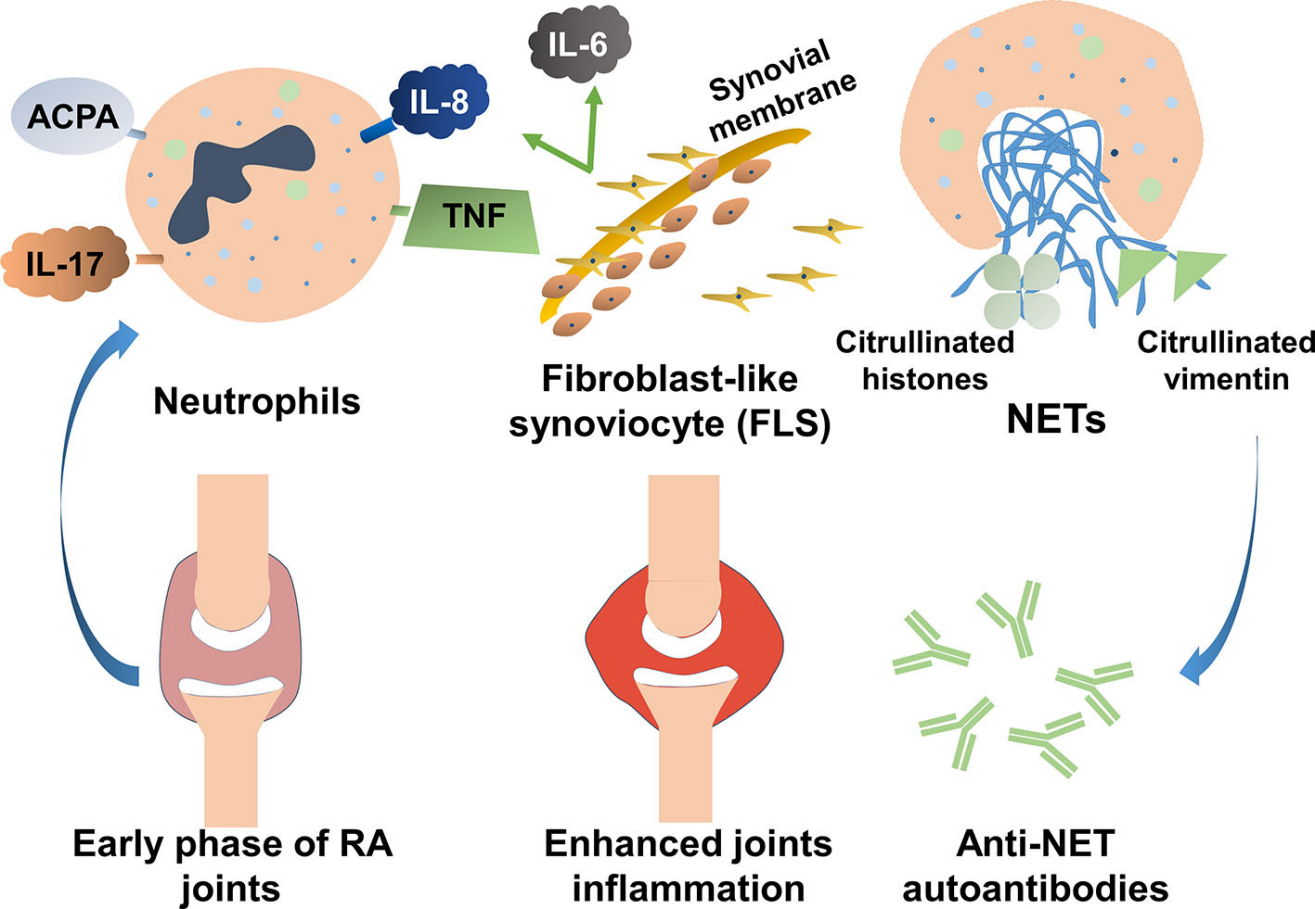
The role of neutrophil extracellular traps (NETs) in the pathogenesis of rheumatoid arthritis (RA). Various elements in the peripheral blood of patients with RA can stimulate NET formation. Excessive NET formation leads to the production of deaminated antigens such as citH2A, citH2B, and citH4 histones. In a joint with RA, presented citrullinated antigens induce antigen-driven autoimmune responses and lead to the generation of anti-NET autoantibodies. Thus, persistent inflammation of the synovial membranes occurs.

Various elements in the peripheral blood of patients with RA, such as autoantibodies or immunostimulatory molecules, reportedly stimulate NET formation (**Table 1**). Excessive NET formation leads to the production of deaminated antigens such as citH2A, citH2B, and citH4 histones (**Figure 1**). Furthermore, NET-borne citrullinated vimentin is a pivotal autoantigen that stimulates the secretion of proinflammatory cytokines (e.g., TNF-α and IL-1) and the expression of PADI4 and receptor activator of nuclear factor kappa B ligand (RANKL) in fibroblast-like synoviocytes (FLSs) (44). FLSs, key effector cells of inflammation in RA, produce multiple cytokines that cause damage in the joints (45). In a joint with RA, presented citrullinated antigens induce antigen-driven autoimmune responses and lead to the generation of anti-NET autoantibodies. Thus, chronic inflammation and autoimmunity exist for a long time. Anti-NET RA recombinant monoclonal antibodies (rmAbs) derived from CD19+ synovial B cells of patients with RA constantly bind NETs.

**Table 1:** Stimuli inducing neutrophil extracellular traps (NETs) formation.

Pathogen triggers	Endogenous triggers	Inflammatory triggers
Bacteria Viruses Fungi Protozoa	TLR 4 FcγRIIIb (37) IL-8, IL-17, TNF-α (38) IFN-γ (39) Calcium salt crystals Urate crystals	Antibodies (40) Immune complexes (19) Lipophosphoglycans (41) M1 protein (42) LPS (8) Hydrogen peroxide (10) PMA (6) Calcium ionophore A23187 (43)

The immunoreactivity of NET-Ags depends on somatic hypermutation (SHM) within the Ig variable H (VH) and variable L (VL) chains of synovial B cells. Moreover, Fab-N-linked-glycosylation determines the reactivity of the autoantibodies (46). Rheumatoid factor (RF), anti-citrullinated protein antibodies (ACPAs), and other autoantibodies in peripheral blood or synovial fluid robustly support NET formation in RA (47). IgG or IgM collected from peripheral blood or synovial fluid of patients with RA induce more NET formation than antibodies from healthy controls (38). Recently, NET-derived elastase results in cartilage matrix disruption and induction of membrane-bound peptidylarginine deiminase-2 released by FLSs. Cartilage fragments are subsequently citrullinated and presented to antigen-specific CD4+ T cells (48). In the NOX/ROS pathway, PAD4-induced histone citrullination promotes chromatin decondensation and NET formation (49). The chromatin-associated protein DEK regulates the structure of extracellular chromatin (50, 51). In models of RA (51), NET formation and protein citrullination are shown to be prevented by depletion of DEK or administration of DEK-targeted aptamers. Both strategies alleviate the symptoms of RA.

In patients with RA, IL-8, IL-17A, and TNF-α reportedly induced NET formation (38). Upon exposure to IL-17A, neutrophils in RAlead to NET formation when the cells are primed with TNF-α. Correlations with NET formation have also been detected for a higher serum level of C-reactive protein (CRP) and a higher erythrocyte sedimentation rate. Furthermore, histone citrullination alone with NET formation can be triggered by the treatment of neutrophils with supernatants harvested from IL-15-stimulated CD69(+)CD8(+) T cells, leading to the extracellular release of citrullinated proteins (52). Conversely, immune complexes induce “incomplete” NET formation (53, 54). Ribonucleoprotein-containing immune complexes induce NET formation depending on mitochondrial ROS rather than NOX (55, 56), which correlates with hypercitrullinated proteins (57) and production of IFN by plasmacytoid dendritic cells (58).

### Signal Transduction Pathways Correlating With NETs

Several underlying signal transduction pathways may promote NET formation in RA and the molecular mechanisms may be pleomorphic. In RA, high concentrations of NE, MPO, PAD4/DNA-complex, and ROS production correspond to the elevated formation of NETs (31, 59).

In neutrophils, Rac is a subunit of the NOX complex (60). Guanosine exchange factor (GEF) activator, Vav, and the p21-activated kinases (Paks) are involved in Rac signaling pathway (61). NOX is indispensable for oxidative burst-dependent NET formation (60–62). Inhibition of NOX reduces NET formation and induces non-canonical NETs (10). ROS are related to the lytic NET formation (63) and stimulate the activation of NE. NE and MPO are also released from azurophilic granules into the nucleoplasm (25). In the nucleus, NE proteolytically cleaves histones and thus interferes with the dense package of chromatin (27). Many physiological and artificial stimuli can activate the MPO-NE pathway (22).

In the peripheral blood of patients with RA, ACPAs stimulate neutrophils to release PAD enzymes (64). *Porphyromonas gingivalis* and smoking are known risk factors for RA, and overexpression of endogenous or bacterial PAD enzymes drives citrullination (65, 66). PAD4 depends on Ca^2+^ (67) and is activated *via* the ROS pathways to convert internal arginine to citrullines (8, 9). Upon PAD4 activation, locally released citrullinated histones enhance the generation of highly mutated clonal B cells resulting in the generation of high-affinity ACPAs (68). At a high titer, fibrinogen citrullinated by PAD 4 acts as the preferred targets for ACPAs (69). Additionally, human leukocyte antigen (HLA)-DR bound PAD4 is recognized by T cells and further contributes to the production of antibodies responded to citrullinated proteins, such as ACPAs and anti-PAD antibodies (70). Anti-PAD4 antibodies have been reported to be closely related to ACPAs (71–73). Kolfenbach et al. evaluated the prediagnosis serum samples of 83 patients with RA and found that 15 RA samples had anti-PAD4 antibodies with a high specificity of 98.8% (71). Interestingly, Erika Darrah et al. first detected PAD4-specific CD4+ T cells in peripheral blood mononuclear cells (PBMCs) of RA patients and found that protease granzyme B (GrB) induced structure changes of PAD4 and promoted the presentation of CD4+ T cell epitopes (74). Overall, further studies are need to demonstrate correlation between PAD4 and RA citrullinome.

Deficiency or inhibition of PADI4 reduces the formation and the size of NETs and alleviates arthritis symptoms in many models, except the murine K/BxN model (75). These findings suggest that PADI4 acts downstream of ROS in NET formation and generates autoantigens that amplify the inflammatory response that precipitates in the pathogenesis of RA (32, 76, 77). PADI4 thus participates in the initiation rather than the effector phase of RA. PAD4, which is associated with histone deamination, can catalyze hypercitrullination by immune-mediated membranolytic pathways (57, 78). Interestingly, in a TNF-induced model of citrullination and arthritis, protein citrullination is executed by PAD2 instead of PAD4. PAD2 is not associated with NET formation (79). Relatively high activity of putatively neutrophil-derived PAD4 has been reported in RA synovial fluid (80). The pathogenesis of RA is also related to T cells specific for citrullinated epitope (81). In brief, the NOX pathways and PAD4 activity can be regarded as critical elements that regulate NET formation and generation of citrullinated autoantigens in RA (82).

### NETs Promote Autoantibody Production, Cytokine Expression, and Cell Activation

Citrullinated components of NETs often serve as self-antigens recognized in the serum of patients with RA (80). Aberrant NETs may promote the externalization of citrullinated autoantigens and immunostimulatory molecules, which enhances the expression of epitopes related to the pathogenesis of RA (38). In RA or osteoarthritis (OA), the levels of IL-6 and IL-8 are upregulated in the presence of NETs, resulting in the activation of FLSs (38, 83). LL-37/DNA complexes induce NETs that activate plasmacytoid dendritic cells *via* TLR7 and TLR9 to produce type I IFN (84). Moreover, NETs are abnormally accumulated in some patients with SLE due to the DNase I inhibitory factors (85, 86), leading to IFN-a release. IFN-a not only enhances NETosis but also induces activation of autoreactive T- and B cells to synthesize autoAbs, such as anti-dsDNA, anti-HNP, and anti-LL37 autoAbs. Moreover, NETs can trigger the production of IL-1β and IL-18, and further stimulate NETosis. These vicious cycles contribute to the imbalance immune homeostasis of SLE. Similarly, NETs are involved in multiple sclerosis (MS). NETosis secretion of antimicrobial proteins induces elevated T-cell activation resulting in tissue damage in MS (87).

NETs triggered by microscopic cholesterol crystals also take part in atherosclerosis (88). NETs induce the activation of leukocytes, platelets, and endothelial cells and further lead to endothelial dysfunction (89). Moreover, NETs promote the production of IL-6 and pro-IL-1β in macrophages (49). Accordingly, these increased cytokines accelerate T helper 17 (TH17) cells differentiation and subsequently induce immune cell recruitment in atherosclerotic lesions. Neutrophils infiltration of culprit lesions results in plaque rupture and erosion *via* NETs (90, 91). Very recently, significantly higher plasma levels of NETs are observed in the carotid lesion site (CLS) of stroke patients. NETs decorated with phosphatidylserine (PS) are detected in thrombi. NET formation requires the synergy of CLS plasma and activated platelets (PLTs). PS-bearing NETs can induce the formation of thrombin and fibrin as well as the conversion of endothelial cells to a procoagulant phenotype (92). These findings indicate that NETs are indispensable in the pathogenesis of many diseases, such as RA, SLE, MS, atherosclerosis, and stroke *via* multiple molecular mechanisms.

### NET and Citrullinated Autoantigens Form a Vicious Cycle in RA

In RA, neutrophils infiltrate synovial tissue, rheumatoid nodules, and the skin (38), when neutrophils form NETs, deaminate proteins, and initiate ACPA production (57). Furthermore, circulating low-density granulocytes (LDGs) in patients with RA tend to increasingly form NETs (19). FLSs activated by NETs express IL-17A, TNF-α, and IL-8 and infiltrate the cartilage, where they enhanced proinflammatory responses (10). The enhanced release of inflammatory cytokines from FLSs driven by NETs causes joint damage and further worsens the condition (31). The secretory leukocyte protease inhibitors can prevent proteolytic maturation of cytokines related to NET formation. Skin lesions may be associated with the insufficient activity of the secretory leukocyte protease inhibitors (93). Importantly, these cytokines trigger the vicious cycle of NET formation and autoantibody biogenesis (38). IL-8 and IL-17 recruit neutrophils and promote the exposure of autoantigens (12, 94). Therefore, NET formation plays a critical role in the pathogenesis of RA. A comprehensive understanding of the mechanisms involved in NET formation may help us develop new therapies *via* targeting NETs to treat NET-related diseases.

## NETs are Associated with RA in Mouse Models

### Neutrophils Drive the Inflammation of Murine Arthritis

RA is an autoimmune disease characterized by progressive destruction of joints. The pathogenesis of RA is still elusive. Researchers usually establish murine models to analyze the pathogenetic sequelae of RA (95). Daisuke and colleagues established an experimental model of male BALB/cAnNCrj (BALB/c) mice injected with an anti-type II collagen antibody and LPS (95). Histological analysis showed that neutrophils were the vast majority of infiltrating cells in the joint space. To determine the effect of neutrophils on arthritis, monoclonal antibodies (mAbs) against Gr-1 (the RB6-8C5 mAb) were intravenously injected into arthritic mice to deplete circulating neutrophils. These experiments suggested that neutrophils are indispensable for the development of arthritis. It is commonly believed that neutrophils play a key role in inflammatory diseases due to their secretion of cytotoxic products (4). However, neutrophils are now considered to be not only effectors of the innate immune systems but also key players in the regulatory circuits of the immune system (96). FcγRs activate neutrophils and trigger a series of signaling events, including ROS generation, protease release as well as the production of chemokines and cytokines. These mediators recruit additional neutrophils and regulate the functions of other immune cells. Hence, they participate in the regulatory network and interplay of innate and adaptive immunity (97). Neutrophils isolated from patients with RA functionally differ from those from healthy controls. Blood- and synovial fluid-derived neutrophils from patients with RA trigger ROS production and display enhanced NET formation (38).

### The Role of NETs in the Etiopathogenesis of RA

NETs are considered to contribute to the pathogenesis of RA (9). Degradation or citrullination of histones driven by PAD4 promotes chromatin decondensation and NET release (38). Furthermore, PAD4 exacerbates inflammatory arthritis and is crucial in some pathways of NET formation (79). Compared to wild-type mice, PADI4 (encoding PAD4)-deficient mice induced by glucose-6-phosphate isomerase showed less severe inflammatory arthritis and reduced autoantibody titers (75). Similarly, in murine collagen-induced arthritis (CIA), inhibition of PADI4 reduced the formation of NETs and arthritis relief (98). However, PAD4 was dispensable in spontaneous arthritis in the K/BxN mouse model (99). NET formation and arthritis in the murine TNFα-induced inflammatory arthritis were investigated to identify the roles of PAD2 and PAD4 for citrullination. PAD2 mediated TNFα-induced citrullination and arthritis but was dispensable for NET formation (99). PAD4, which is involved in NET formation, was dispensable for citrullination. These studies indicate that various pathogenic pathways may cause murine arthritis. These can be dependent or independent of NETs.

Further evidence supported the roles of NETs in the pathogenesis of RA that blocked NET formation and protein citrullination was caused by treatment with DEK-targeted aptamers, as DEK is essential for certain pathways of NET formation (38, 100). Autoantibodies that recognize DEK have been detected in the sera of patients with autoimmune diseases, such as systemic lupus erythematosus (SLE) and adolescent idiopathic arthritis (JIA) (100). DEK acts as a chemoattractant, triggers inflammatory responses, and plays an important role in a murine model of arthritis. Aptamers targeting DEK could reduce NETs formation, slow the progression of joint inflammation, and ameliorate the disease symptoms in arthritic mice (51).

Another established murine model of RA is the K/BxN mice. The pathology is similar to that of human RA. The K/BxN mice are generated by crossing KRN-C57BL/6 mice, which carry a transgenic T cell receptor, with autoimmunity-prone non-obese diabetes (NOD) mice (101). K/BxN mice develop IgG autoantibodies against glucose-6-phosphate isomerase, which precipitate joint damage.

Mice lacking functional NOX have the further aggravation of arthritic symptoms. ROS suppression occurs in patients with chronic granulomas disease (CGD) due to impaired function of NOX (35). This implies that ROS in NETs are derived from additional mechanisms beyond the NOX pathway (86). NET formation triggered by nicotine (102) was found to be dependent on mitochondrial ROS rather than depend on NOX (20). However, Cl-amidine, a PAD inhibitor, did not inhibit the formation of mitochondrial ROS but inhibit NETs in the New Zealand mixed 2328 (NZM) murine mice (34).

## Diagnosing RA with NETs

Currently, the laboratory diagnosis of RA relies on the detection of RF and ACPAs (59, 103). These autoantibodies can be found in most RA patients, and the titer of ACPAs correlates with the severity of RA (4). Although many autoantibodies markers have been applied for patients’ diagnosis with RA, ACPAs are the most disease-specific markers with the highest specificity and sensitivity (103). Khandpur et al (38). analyzed 55 Patients with RA and 36 healthy volunteers or patients with OA. The results showed that NET formation was associated with the levels of ACPAs and indicated NETs were a potential target for ACPAs. ACPAs include antibodies targeting keratin (AKA), perinuclear factor (APF), profillagrin/fillagrin (AFA), Sa, and artificial cyclic citrulline peptide (CCP). The diagnostic specificity of four kinds of ACPAs (APF, AKA, AFA, and CCP (II)) for RA was more than 90%, which is significantly higher than that of RF (77.7%), suggesting that ACPAs can be employed as effective diagnostic antibodies for RA. However, the sensitivity of ACPAs for RA differs due to differences in antigens preparation and detection methods (104–106).

Recently, some studies have focused on the detection of potential signaling pathways that lead to the increase of NET formation in RA. This is to determine whether the products of NET formation are useful for diagnosis. NETs as target biomarkers have been reported in many autoimmune diseases. Levels of human neutrophil peptide 1–3 (HNP 1–3), a part of NETs, were found to be significantly higher in patients with lupus nephritis (LN) than in healthy controls. They are an independent indicator of LN [P = 0.006, odds ratio (OR) = 7.5, 95% confidence interval (CI), 1.782–31.842]. Moreover, the NET-inducing capacity might be a novel biomarker of ANCA-associated vasculitis (AAV). The levels of NET degradation products, such as circulating free DNA (cfDNA), free nucleosomes, NE-DNA, and MPO-DNA complexes, are reportedly increased in patients with RA (3). NET-derived products were previously analyzed *in vitro* by microscopy and enzyme-linked immunosorbent assay (ELISA). Receiver operating characteristic (ROC) curves showed spontaneously increased formation of NETs and production of ROS in patients with RA (59). NET-derived cell-free nucleosomes in RA serum showed diagnostic value with an area under the ROC > 97% with 91% sensitivity and 92% specificity (59). No significant differences were detected between ACPA-positive and ACPA-negative patients (59). Recently, the level of anti-NET antibodies (ANETA) in RA serum was reported to be significantly higher in rheumatoid factor-positive than that in seronegative patients (107). The collective evidence suggests that the quantitative detection of the NET-derived products may be a useful complementary tool to identify individuals at risk and to monitor patients with RA.

## Therapeutic Targeting of NET Formation to Treat RA

### Clearance of NETs

DNAse-1 dismantles NETs *in vitro*. *In vivo*, DNase-1 does not interfere with NET production, but fragments the DNA and destroys the backbone of the NETs (9). DNase-1 reportedly promotes the escape of group A *Streptococcus* (GAS) from being killed by NETs. Enhanced neutrophil depletion of GAS and reduced virulence occur in the presence of the DNase I inhibitor G-actin (108). Recently, several studies have reported that bacterial DNases degrade NETs, allowing the bacteria to escape killing in NETs (109–111). However, Bryan et al. injected *S. aureus* intraperitoneally into mice and monitored the infections with minimally invasive nonsurgical luminescent imaging, showing that DNase reduced bacterial growth (112). Kolaczkowska and colleagues also demonstrated that DNase effectively eliminated NET-borne DNA and inhibited the proteolytic activity of NE (25). Nevertheless, NETs still show some antimicrobial activities, as most of the histones remained. The circulating zymogen form of factor VII activating protease (FSAP) can be activated by histones and the nuclear lobules of NETs. NETs bound to FSAP fail to activate pro-FASP. However, histones release after the degradation of NETs by DNase dramatically stimulating pro-FASP activity (113). Pathogenic micro-organisms produce DNases that inhibit the generation of ROS in the later stage and lead to escape the killing in NETs (114). In addition, NETs are cleared *via* phagocytosis of macrophages, which increases the release of cfDNA (115). Whether the pathogenesis of RA involves macrophage dysfunction needs further examination. The cfDNA levels in synovial fluids were correlated with neutrophil counts but not with macrophage counts (80). The increased NETs levels in RA synovial fluids may be caused by either impaired activity of DNase-1 or by inhibitors of DNase-1. Serum DNase-1 activity is negatively correlated with inflammatory markers and neutrophil counts, suggesting that insufficient DNase-1 activity is an important factor in the regulation of NET persistence. The elevated cfDNA levels in the synovial fluid may be an important source of “altered self.” Only very few eosinophils and mast cells exist in the synovial fluid of patients with RA, suggesting that cfDNA are mainly derived from NETs. An advantage of DNase-1 is low toxicity, which has been verified in murine models of breast cancer (116), lupus (117), or lung damage (118). Exogenous administration of DNase I may be used to dismantle NETs and can, therefore, be considered for the treatment of RA.

### Inhibition of NET Formation

Additionally, drugs that reduce the formation of NETs may also be used to treat and relieve RA. Delivery of the NE inhibitor sivelestat *via* a nanoparticle system to LPS-induced endotoxin shock mouse model inhibits NET formation, reduces circulating NE, and prevents mice from endotoxic shock (119). CI-amidine can reduce protein citrullination in the pGIA mouse model (120). Rituximab and belimumab reduce NET formation by blocking the immune-complexes formation (121). Emodin accelerates apoptosis and suppresses autophagy and NET formation by reducing IL-6, IFN-γ, and TNF-α in the murine adjuvant-induced arthritis (AA) (122). Polydatin (PD) reduces NET formation of bone marrow-derived neutrophils and in patients with RA. Similarly, in CIA mice, the deposition of NETs in the ankle joints is decreased by PD-treatment (123). Ascomycin and cyclosporine A can decrease IL-8 induced NET formation by inhibiting the calcineurin pathway (124). Triptolide (TP) exhibits potential as an RA therapeutic by lowering neutrophil recruitment and downregulating the expression of TNF-α and IL-6. TP is also able to suppress NET formation and autophagy of neutrophils (125). Moreover, celastrol can inhibit NOX-dependent NET formation (126). Tocilizumab likewise shows the potential to reduce autoantibody levels and, consequently, immune complex formation in patients with RA (127). Nevertheless, there are differences in the immune system and physiological function between murine models and humans. Given the limited scope of current clinical standard therapies, more clinical research is required to establish NET reducing therapies.

## Perspectives and Conclusion

The accumulated data on the role of NETs in RA has brought NETs into focus as novel therapeutic targets for RA. The future will tell whether a blockage in NET formation or increased NET catabolism will win the race.

## Author Contributions

All authors listed have made a substantial, direct, and intellectual contribution to the work and approved it for publication.

## Funding

This work was partially supported by the German Research Foundation (DFG) (TRR241: B04; CRC1181: C03, Z02; FOR 2886 projects B03. This work was funded by the National Natural Science Foundation of China [grant number 81501412].

## Conflict of Interest

The authors declare that the research was conducted in the absence of any commercial or financial relationships that could be construed as a potential conflict of interest.
